# Gene-Expression Analysis Identifies IGFBP2 Dysregulation in Dental Pulp Cells From Human Cleidocranial Dysplasia

**DOI:** 10.3389/fgene.2018.00178

**Published:** 2018-05-23

**Authors:** Stephen L. Greene, Olga Mamaeva, David K. Crossman, Changming Lu, Mary MacDougall

**Affiliations:** ^1^Department of Pediatric Dentistry, School of Dentistry, The University of Alabama at Birmingham, Birmingham, AL, United States; ^2^Institute of Oral Health Research, School of Dentistry, The University of Alabama at Birmingham, Birmingham, AL, United States; ^3^Department of Genetics, School of Medicine, The University of Alabama at Birmingham, Birmingham, AL, United States; ^4^Faculty of Dentistry, University of British Columbia, Vancouver, BC, Canada

**Keywords:** cleidocranial dysplasia, RUNX2, IGFBP2, IGF, dentinogenesis

## Abstract

Cleidocranial dysplasia (CCD) is an autosomal dominant disorder affecting osteoblast differentiation, chondrocyte maturation, skeletal morphogenesis, and tooth formation. Dental phenotype in CCD include over-retained primary teeth, failed eruption of permanent teeth, and supernumerary teeth. The underlying mechanism is unclear. We previously reported one CCD patient with allelic *RUNX2* deletion (CCD-011). In the current study, we determined the transcriptomic profiles of dental pulp cells from this patient compared to one sex-and-age matched non-affected individual. Next Generation RNA sequencing revealed that 60 genes were significantly dysregulated (63% upregulated and 27% downregulated). Among them, *IGFBP2* (insulin-like growth factor binding protein-2) was found to be upregulated more than twofold in comparison to control cells. Stable overexpression of RUNX2 in CCD-011 pulp cells resulted in the reduction of *IGFBP2*. Moreover, *ALPL* expression was up-regulated in CCD-011 pulp cells after introduction of normal RUNX2. Promoter analysis revealed that there are four proximal putative RUNX2 binding sites in -1.5 kb *IGFBP2* promoter region. Relative luciferase assay confirmed that *IGFBP2* is a direct target of RUNX2. Immunohistochemistry demonstrated that IGFBP2 was expressed in odontoblasts but not ameloblasts. This report demonstrated the importance of RUNX2 in the regulation of gene profile related to dental pulp cells and provided novel insight of RUNX2 into the negative regulation of IGFBP2.

## Introduction

Cleidocranial dysplasia (CCD, OMIM #119600) is a rare autosomal dominant genetic disorder characterized by bone and tooth abnormalities. Classical skeletal abnormalities seen in CCD include wormian bones, underdeveloped or completely absent clavicles, frontal and/or parietal bossing, flat nasal bridge, hypertelorism, late closure of the sutures and frontals of the skull, and short stature (Mundlos, 1999). CCD dental abnormalities include delayed tooth eruption, over-retained primary teeth and supernumerary teeth.

CCD is majorly caused by mutations in the transcription factor RUNX2 (runt-related transcription factor 2) : ∼70% of CCD cases are due to heterozygous *RUNX2* mutations, less than 10% of cases due to *RUNX2* copy number variations, and remaining cases by unknown etiology. RUNX2 is one of three members of the RUNX gene family, a master regulator important for osteoblast differentiation, chondrocyte maturation, skeletal morphogenesis, and odontogenesis (Cohen, 2009, 2013). Runx2 possesses several active domains such as the transactivation domains, glutamine/alanine rich domain, runt homology domain, nuclear localization signal, proline/serine/threonine rich domain, nuclear matrix targeting signal, repression domain, and VWRPY region (Vimalraj et al., 2015). Upon various stimuli, Runx2 interacts with different proteins resulting in positive or negative regulation of its target genes. Mutations in different domains may distinctly affect RUNX2 function in the transcriptional regulation of its target genes, which is reflected by different phenotypes seen in CCD.

Tooth development is a very complex process, which involves many transcription factors and signaling networks to ensure an ordered and controlled development of tooth germs and dentition. RUNX2 is reported to be important for tooth formation. RUNX2 has been reported to be involved in the regulation of dentin sialophosphoprotein (Chen et al., 2005), one of the principal proteins of the dentin extracellular matrix. Furthermore, RUNX2 regulates the alveolar remodeling process essential for tooth eruption and may play a role in the maintenance of the periodontal ligament (Camilleri and McDonald, 2006). Dental abnormalities seen in CCD patients may be a direct result of RUNX2 dysfunction in tooth-forming cells. Thus, it is necessary to comprehensively identify the targets of RUNX2 in dental cells. In this study, we analyzed the global transcriptomic profile of dental pulp cells isolated from a patient (CCD-011) with heterozygous novel microdeletion encompassing the entire RUNX2 locus and a segment of SUPT3H in comparison with age- and sex-matched pulp cells (Greene et al., 2018). Over 25,000 genes involved in important biological pathways were evaluated in order to identify novel RUNX2 target genes using next-generation RNA sequencing. For the first time, we identified IGFBP2 as a direct target of RUNX2 in dental pulp cells.

## Materials and Methods

### Cell Culture

Human study protocols and patient consents were reviewed and approved by the Institutional Review Board at the University of Alabama at Birmingham. CCD-011 dental pulp cells and age- and sex-matched control dental pulp cells from non-affected healthy individual were grown in αMEM supplemented with 10% FBS, ascorbic acid (50 μg/ml), penicillin (100 U/ml), and streptomycin (100 μg/ml) at 37°C with 5% CO2. For cell differentiation, cells were cultured with growth media above supplemented with 10 mM β- glycerophosphate for indicated time.

### Next Generation RNA Sequencing

Total RNA from CCD-011 and control pulp cells were isolated using RNeasy Mini Kit (Qiagen) according to the manufacturer’s protocols. Next generation RNA Sequencing (RNA-Seq) was performed at the Heflin Center for Genomic Sciences Genomic Core. Briefly, mRNA sequencing was performed on Illumina HiSeq2000 platform. Total RNA was assessed using the Agilent 2100 Bioanalyzer followed by 2 rounds of poly A+ selection and conversion to cDNA. The TruSeq RNA Library Prep Kit were followed according to the manufacturer’s instructions (Illumina, San Diego, CA, United States). Library construction consisted of random fragmentation of the polyA mRNA, followed by cDNA production using random primers. The ends of the cDNA were repaired and A-tailed, and adaptors were ligated for indexing (up to 12 different barcodes per lane) during the sequencing runs. The cDNA libraries were quantitated using quantitative PCR in a Roche LightCycler 480 with the Kapa Biosystems kit for library quantitation (Kapa Biosystems, Woburn, MA, United States) prior to cluster generation. Clusters yielded approximately 725K–825K clusters/mm^2^. Cluster density and quality was determined during the run after the first base addition parameters were analyzed. Paired end 2 × 50 bp sequencing runs were run to align the cDNA sequences to the reference genome. Because CCD sample with allelic RUNX2 deletion is very rare, we have collected one sample (CCD-011) so far which was used in this experiment. The data has been deposited to the sequence read archive^1^.

### RNA-Seq Data Analysis

Image files from the sequencer were converted to raw sequence fastq files using the Illumina compute server running CASAVA version 1.8.2. Quality control of fastq files was checked with FastQC version 0.10.1 and found that no trimming or removal of poor quality sequences was needed. Alignments of the raw sequence reads to the UCSC human hg19 reference genome was performed using TopHat version 2.0.9 with the following parameters: –library-type fr-unstranded; -r 150. Transcript abundances was calculated using Cufflinks version 2.1.1 with the following parameters: -g; -b; -u. Cuffmerge was then used to merge the two transcript abundance files together, followed by pairwise differential expression with Cuffdiff (parameters used: -u; -b).

### Ingenuity Pathway Analysis

The gene lists from Cuffdiff were uploaded to Ingenuity Pathway Analysis and the Core Analysis was used to identify significant interactions, downstream effects, and pathways.

### Real-Time PCR

Total RNA were isolated from indicated cells as described as above. Single-strand cDNA was synthesized using the High-Capacity cDNA Reverse Transcription Kit (Applied Biosystems, Foster City, Calif., United States). Primer sets for *IGFBP2, GREM1* (Gremlin 1, DAN Family BMP Antagonist), *BARX1* (BarH-like homeobox 1), and *ALPL* (alkaline phosphatase, liver/bone/kidney) were purchased from the Integrated DNA Technologies (IDT, Coralville, Iowa., United States). Other primer sets were synthesized by IDT as follows: TFAP2A(transcription factor AP-2 alpha)-For: 5′-GAGCCATGGCACGCACGAGACGGTATCTA-3′, TFAP2A-Rev: 5′-GAGCTCGAGCTCGCAGTCCTCGTACTTGA-3′; LYPD6B (LY6/PLAUR Domain Containing 6B)-For: 5′-GTTTCCTGACCCGTGAAATG-3′, LYPD6B-Rev: 5′-GTCCCGTCCAGATGTTGG-3′; RUNX2-For: 5′-TTACTTACACCCCCCAGTC-3′, RUNX2- Rev: 5′-CACTCTGGCTTTGGG AAGAG-3′;and endogenous control GAPDH-For: 5′-AGGTCGGAGTCA ACGGATTTG-3′, GAPDH-Rev: 5′-GGGGTAACTGTGC-CTATTCG-3′. Quantitative PCR using SYBR Green SuperMix (Qiagen) was performed as we previously described (Lu et al., 2014). The level of mRNA expression was measured using threshold cycle (CT) according to the ΔΔCT method (Livak and Schmittgen, 2001).

### Establishment of CCD-011 Dental Pulp Cells With Stable Over-Expression of RUNX2

Recombinant pLenti-RUNX2 was customarily generated by VectorBuilder (Santa Clara, CA) by inserting normal full length of human RUNX2 into lentiviral expression empty vector. Lentivirus were prepared by transfection of expression vector, either pLenti-RUNX2 or Lentiviral empty vector together with packaging vectors (pMD2.G and psPAX.2; Addgene, Cambridge, MA, United States) into HEK-293T cells. The viruses were concentrated and titered. CCD-011 dental pulp cells were infected with empty lentivirus (lenti-Con) or recombinant lentivirus (lenti-RUNX2), respectively at the MOI of 0.5. Cells were further selected using G418 (20 μg/ml) for 1 week.

### Luciferase Report Assay

A 1.5 kb fragment of the proximal IGFBP2 promoter was amplified with the following primer set: IGFBP2-For: 5′-CAGGGTACCCTGTGCCCTTGCTAACCGCCCATTTC-3′, IG FBP2-Rev: 5′-CAGGCTAGCCGGGTCCTAAGGGCCGGCTTCTCC-3′ (restriction enzyme site KpnI and NheI were underlined). The DNA fragment was inserted into the luciferase reporter vector pGL4.18 [*luc2P*/Neo] (Promega Corporation, Madison, Wisconsin, United States) by KpnI and 3′ NheI (IGFBP2-pGL4.18). For luciferase reporter assay, 5 × 10^4^ HEK293T cells were cultured in 12-well plates overnight. Next day, cells were transfected with 1 μg of with lentiviral empty vector or lenti-RUNX2 together with 20 ng of IGFBP2-pGL4.18 and *Renilla* luciferase pGL4.74 [*hRluc*/TK] plasmid (Promega, Madison, WI, United States) using PolyJet^TM^ In Vitro DNA Transfection Reagent (SignaGen Laboratories, Rockville, MD, United States). At 48 h post transfection, luciferase activity in each well was measured by Dual-Luciferase^®^ Reporter Assay System (Promega) and normalized to Renilla.

### Immunohistochemistry

Histological sections (5 μm) of postnatal day 5 mice tissue samples were prepared for immunohistochemistry as previously described (MacDougall et al., 1998). Immunostaining was performed using primary antibodies against IGFBP2 (Cell Signaling Technologies, Danvers, MA, United States). After deparaffinization, heat induced citrate antigen retrieval and blocking with 3% goat serum, 1% bovine serum albumin, and 0.5% tween in phosphate-buffered saline for 20 min at RT, mouse tooth sections were incubated with primary antibody for 1 hour at room temperature, followed by horseradish peroxidate (HRP) poly conjugate for 10 min and 3,3′-diaminobenzidine for optimal time. Images were visualized and captured by Nikon Eclipse TE2000-E microscope (Nikon Instruments, Melville, NY, United States).

## Results

### Dysregulation in Gene Expression in CCD-011 Dental Pulp Cells

In order to identify novel *RUNX2* target genes involved in tooth formation and signaling pathways that potentially contribute to the dental phenotypes seen in CCD, Next-Generation RNA Sequencing was performed on CCD-011 dental pulp cells carrying a total *RUNX2* deletion in one allele and compared to one age- and sex-matched control pulp cells. Of the 25,643 genes analyzed, 11,039 genes had no detectable signal in both CCD and control samples tested, leaving 14,604 genes that were evaluated for differential gene expression. In the detectable genes, 60 transcripts (4.1%) were found to be statistically significantly dysregulated with 63% upregulated and 27% downregulated (fold change ≥2; *q*-value < 0.05). The top 10 genes with differential change are listed in **Table 1**. Analysis of isoform differential expression revealed 35% downregulated and 65% upregulated transcripts. The isoforms with twofold statistically significant change are listed in Supplementary Table 1. Ingenuity Pathway Analysis revealed top up- and down-regulated genes delineating multiple putative RUNX2 targets genes both upstream and downstream (Supplementary Figures 1, 2).

**Table 1:** Top 10 up- or downregulated genes in CCD-011 dental pulp cells in comparison to control cells.

Folds	Upregulated genes	Folds	Downregulated genes
42.3	Thioredoxin Interacting Protein	–104.0	Pregnancy Specific Beta-1-Glycoprotein 4
25.2	Insulin-like growth factor binding protein	–35.5	Claudin 11
17.1	Proenkephalin	–33.7	ALPL Alkaline Phosphatase
16.6	Plasminogen Activator, Tissue Type	–16.4	Early Growth Response 1
14.7	Inhibitor Of DNA Binding 1, HLH Protein	–9.7	Spondin 2
12.6	Glypican 3	–8.9	Claudin 1
10.0	Neuroblastoma 1, DAN Family BMP Antagonist	–8.9	Cytochrome P450 Family 1 Subfamily B Member 1
9.4	Inhibitor Of DNA Binding 3, HLH Protein	–8.8	Small Nucleolar RNA Host Gene 5
8.9	Pregnancy Specific Beta-1-Glycoprotein 5	–8.7	Tribbles Pseudokinase 3
7.5	Phospholipase C Beta 4	–7.9	Growth Differentiation Factor 5

**FIGURE 1:**
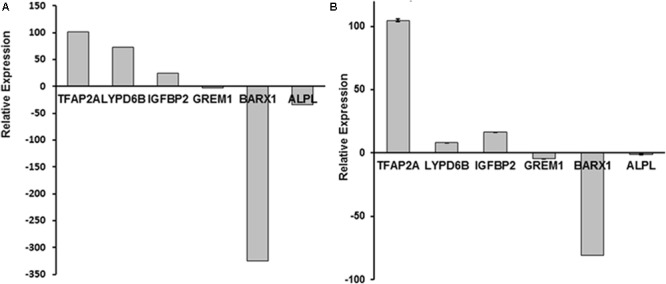
Six dysregulated genes in CCD-011 dental pulp cells. **(A)** Six genes: *TFAP2A, GREM1, BARX1, ALPL, LYPD6B*, and *IGFBP2*, was dysregulated in CCD-011 dental pulp cells identified by RNA-Seq. **(B)** Real-time PCR quantitative analysis for the six genes listed above.

Among 60 genes identified by RNA-Seq, six genes (**Figure 1A**), *TFAP2A, GREM1, BARX1, ALPL, LYPD6B*, and *IGFBP2* have been previously reported to be associated with osteogenesis and/or craniofacial development (Eckstein et al., 2002; Stoetzel et al., 2009; Tekin et al., 2009; Chung et al., 2012; Leijten et al., 2013; Nichols et al., 2013; Gasque et al., 2015). Their dysregulation in CCD-001 pulp cells was further confirmed by qPCR (**Figure 1B**). Four genes: *TFAP2A, GREM1, BARX1, ALPL* have been reported to be involved in craniofacial and/ or tooth development (Milunsky et al., 2008; Mitsiadis and Drouin, 2008; Nagatomo et al., 2008; Li et al., 2013; Liu et al., 2014). However, the remaining two genes, *LYPD6B* and *IGFBP2*, have not been described in tooth development.

### RUNX2 Introduction Partially Rescued the Dysregulated Genes in CCD-011 Pulp Cells

**FIGURE 2:**
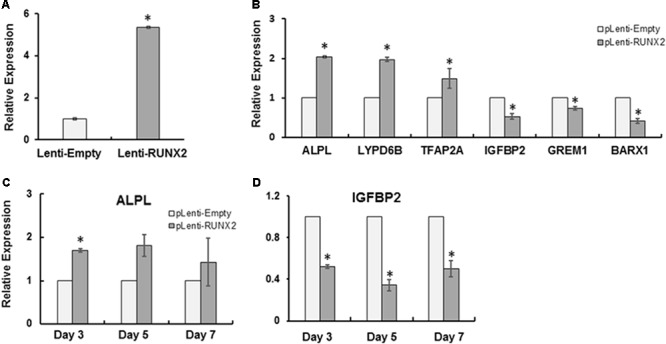
RUNX2 introduction partially rescued the dysregulated genes in CCD-011 pulp cells. **(A)** CCD-011 dental pulp cells with stable overexpression of normal human RUNX2 (Lenti-RUNX2) and control cells (Lenti-Empty). **(B)** Quantitative analysis of human RUNX2 expression in CCD-011 dental pulp cells with Lenti-RUNX2 and Lenti-Empty. **(C)** Quantitative analysis of the expression of indicated six genes in CCD-011dental cells with Lenti-RUNX2 and Lenti-Empty. **(D)** Quantitative analysis of the expression of *ALPL* and *IGFBP2* in CCD-011dental cells under odontoblastic differentiation condition. Quantitatively real-time PCR was performed as detailed in Section “Materials and Methods”. The relative expression of the indicated genes in CCD-011 dental pulp cells with Lenti-RUNX2 was normalized to endogenous control, GAPDH, and compared with the control cells with Lenti-Empty. ^∗^*P* < 0.05 by Student’s *t*-test between the pulp cells with Lenti-RUNX2 and cells with Lenti-Empty. Data were presented as mean ± SD from one representative of two independent experiments.

To investigate the role of RUNX2 in the regulation of target genes involved in CCD, CCD-011 pulp cells were transduced with lenti-RUNX2 to introduce the full length of human RUNX2 (**Figure 2A**). Cells transduced with empty lentivirus was used as control. As seen in **Figure 2B**, there was almost fivefold higher level of RUNX2 in CCD-011 pulp cells transduced with Lenti-RUNX2 in comparison to that in cells transduced with empty lentivirus. To investigate if RUNX2 introduction could rescue the dysregulation of the six genes identified by RNA-Seq in CCD-011 pulp cells, their expression was quantitatively analyzed. As seen in **Figure 2C**, the expression of *TFAP2A, LYPD6B*, and *ALPL* was increased in CCD-011 pulp cells after introducing normal human RUNX2, indicating the positive regulation of their expression by RUNX2. This finding also suggests that their up-regulation in CCD-011 dental cells seen in **Figure 1** may be due to individual variation. However, the expression of *IFGBP2, GREM1*, and *BARX1* was down-regulated in CCD-011 pulp cells with normal RUNX2 introduction. Under differentiation condition, IGFBP2 expression was further down-regulated by RUNX2 overexpression distinct from the up-regulation of ALPL (**Figure 2D**). Taken together, these findings indicate that introduction of normal human RUNX2 gene into CCD-011pulp cells can partially rescue the dysregulation of gene expression in these cells.

### IGFBP2 Is a Direct Target of RUNX2

**FIGURE 3:**
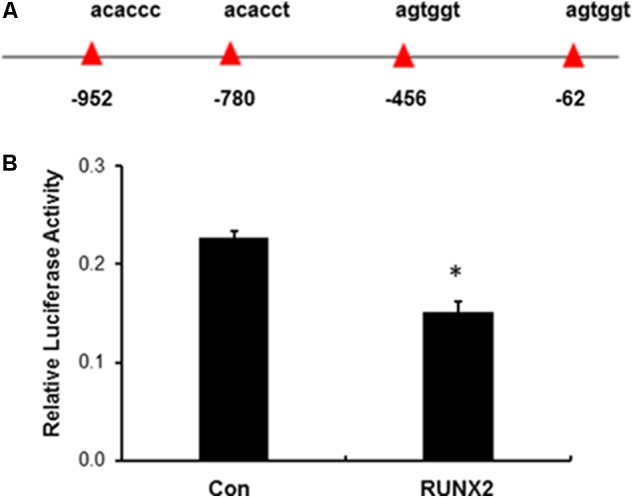
Negative regulation of IGFBP2 by RUNX2. **(A)** Potential RUNX2 DNA binding site in the promoter region of *IGFBP2*. **(B)** Luciferase reporter assay revealed that IGFBP2 was negatively regulated by RUNX2. HEK293T cells in 24-well plates were transfected with empty lentivirus (Con) and lenti-RUNX2 (RUNX2) together with IGFBP2 luciferase reporter vector and Renilla vector followed by measuring the luciferase activity at the method detailed in the Section “Material and Methods”. ^∗^*P* < 0.01 by Student’s *t*-test between two groups. Data were presented as mean+SD from one representative of two independent experiments.

IGFBP2 is a highly conserved family of six IGFBPs that circulate in serum and local biological fluid at relatively high concentrations. IGFBP2 serves as carrier proteins through high binding to IGFs and regulates their bioactivity (Ehrenborg et al., 1991; DeMambro et al., 2008). To determine if RUNX2 directly regulates IGFBP2, the human IGFBP2 promoter was first analyzed for putative RUNX2 binding sites based on previous reports (Ducy et al., 1997; Chen et al., 2002). As shown in **Figure 3A**, in the 1.45 kb segment of the proximal promoter, there are total of four potential RUNX2 binding sites. This promoter fragment was then amplified and inserted into luciferase reporter vector (IGFBP2-pGL4.18). HEK293T cells were transfected with lentiviral empty vector or lenti-RUNX2 together with luciferase IGFBP2-pGL4.18 and Renilla vector for luciferase assay. As shown in **Figure 3B**, there was significant lower level of luciferase activity in HEK293T cells with lenti-RUNX2 compared with that in cells with lentiviral empty vector, demonstrating that IGFBP2 is a direct target of RUNX2.

### Localization and Expression of IGFBP2 in Mouse Molars

**FIGURE 4:**
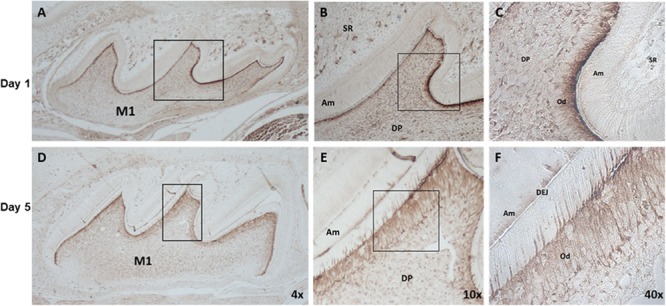
IGFBP2 expression in mouse dental tissues, alveolar bone and skeletal muscle. IGFBP2 expression in dental tissue from day 1 **(A–C)** and day 5 **(D–F)** postnatal mouse revealed by immunohistochemical staining. odontoblast (Od), dental pulp (DP), stellate reticulum (SR), amnioblast (Am), dentino-enamel junction (DEJ), first molar (M1).

To determine the protein expression of IGFBP2 in the dental tissue during tooth development, postnatal day-1 and -5 mouse tooth sections were analyzed by immunohistochemistry. IGFBP2 was detected in skeletal muscle, alveolar bone, the odontoblasts, throughout the dental pulp, and within the oral epithelium (**Figure 4**). Staining was also seen within the stellate reticulum region associated with blood vessels. No staining was detectable within the epithelial components of the tooth including the ameloblasts, stellate reticulum, and outer enamel epithelium. These findings strongly suggest IGFBP2 may play a functionally important role during odontogenesis associated with dentinogenesis.

## Discussion

CCD is primarily caused by RUNX2 functional alteration due to differential mutations or copy number variations. Although extensive efforts have been made in the past decades, it remains unclear how RUNX2 is involved in the pathogenesis of CCD. In this study, using RNA-Seq, we systemically analyzed the gene profiles from the pulp cells from one rare CCD patient with an allelic loss of total RUNX2 and pulp cells from one sex- and age-matched non-affected individual. We found that many genes associated with osteogenesis or dentinogenesis are dysregulated in CCD cells due to RUNX2 haploinsufficiency. For the first time, we found that IGFBP2 was a direct target of RUNX2 and increased in CCD pulps cells, indicating its potential role in the pathogenesis of CCD.

RUNX2 is the master transcriptional factor involved in bone formation through regulation of the expression of many bone matrix genes including osteocalcin, bone sialoprotein, osteopontin, and collagen I. In contrast to the relatively known functional role of RUNX2 in skeletal bone development and maintenance, it remains unclear how RUNX2 is involved in tooth formation. Importantly, RUNX2 may play different roles in tooth formation in humans and mice. Animal studies showed that tooth development was arrested in RUNX2 null mice at the late bud stage and normal in heterozygous RUNX2 mutant mice (D’Souza et al., 1999; Adhami et al., 2015), in contrast to the findings that there are supernumerary teeth seen in CCD patients with RUNX2 mutations or copy variations. The CCD-011 dental pulp cells with allelic deletion of total RUNX2 provided a useful cell tool to investigate how RUNX2 deficiency affects downstream targets and contribute to the dentinogenesis dysregulation related to CCD. Using RNA-Seq, we comprehensively analyzed and compared the different gene profiles between CCD-011 and control dental pulp cells. In this study, we found that numerous gene expressions in CCD-011 pulp cells were up- and downregulated. These genes are associated within a number of biological processes including DNA replication, recombination and repair, cellular movement, assembly and organization, and organ morphology (**Table 1**, Supplementary Table S1, and Supplementary Figures S1, S2), suggesting the critical potential role in tooth development.

IGF-1(insulin-like growth factor 1) and IGF-2 are part of a complex systems and involved in various physiological and pathological processes, including cell differentiation and proliferation, morphogenesis, growth, metabolism, and carcinogenesis (Aguirre et al., 2016; Kasprzak et al., 2017; Mathew et al., 2017; Takahashi, 2017). Accumulating evidences from *in vitro* and *in vivo* studies demonstrated that IGFs are critical in the regulation of odonto/osteogenic differentiation and subsequent tooth/ bone formation (Young, 1995; Mohan and Kesavan, 2012; Ma et al., 2016; Matsumura et al., 2017). Since IGFBPs can bind to IGFs to inhibit or potentiate their bioactivity, it is conceivable that IGFBP2 dysregulation could affect IGFs signaling in the development and maintenance of skeletal and dental tissues, contributing to tooth/bone disorders. The expression patterns and functional role of IGFBP2 in skeletal tissues have been reported. IGFBP2 levels decline during neonatal and pubertal growth and increase with advancing age in humans. IGFBP2 generally inhibits IGF action when added to osteoblastic cells in culture (Conover, 2008). Transgenic mice with overexpression of IGFBP2 exhibit skeletal deficiencies (Eckstein et al., 2002). Clinical studies revealed a potentially deleterious role of IGFBP2 on bone density in aging men and women (Amin et al., 2004). However, the effects of IGFBP2 deficiency in bone development is controversial that maybe influenced by gender, age, and others factors (DeMambro et al., 2008). The functional role of IGFBP2 in odontogenic differentiation and tooth development is freshly reported. Studies in human dental pulp cells (DPCs) showed that IGFBP2 were expressed in DPCs, upregulated during odontogenic differentiation and coordinately regulated IGF-1-induced matrix mineralization with IGFBP-3 (Alkharobi et al., 2016). However, Kim et al. (2012) reported that IGFBP2 was highly expressed on dental epithelium of the initiation stage but declined at bell stage of tooth development and suggested a negative role of IGFBP2 in tooth development. Our current studies showed that IGFBP2 was increased in CCD dental pulp cells with reduction in ALP expression, indicating a potential negative role of IGFBP2 in extracellular matrix mineralization. Further studies, such as using transgenic mice with IGFBP2 overexpression or deficiency, are necessary to determine the actual role of IGFBP2 in odontogenic differentiation and tooth development.

Although RUNX2 has been implicated in both positive and negative regulation of gene expression, it is the first time for us to report the transcriptional regulation of IGFBP2 by RUNX2. The expression and activity of RUNX2 is affected by a diversity of signaling pathways, which include extracellular matrix protein, cell-surface integrin, and growth factors. Previous studies reported that RUNX2 expression, both mRNA and protein levels, is regulated by IGF-1 signaling (Sun et al., 2001). IGF-1 also mediates endogenous RUNX2 activity through a phosphatidylinositol 3-Kinase/ERK-dependent and Akt-independent signaling pathway (Qiao et al., 2004). Since, IGFBP2 is negatively regulated by RUNX2, IGFs-RUNX2-IGFBP2 axis may play important physiopathological role in the tooth/bone development and disorders.

In summary, our studies have revealed a critical role of RUNX2 in the regulation of gene expression pattern associated with CCD dental pulp cells. Importantly, our studies demonstrate that RUNX2 is a negative regulator of IGFBP2, which may be involved in the pathogenesis of human CCD with haploinsufficiency in RUNX2.

## Author Contributions

SG performed the experiments, analyzed the data, and wrote the draft of the manuscript. OM participated in the experiments and the analysis of the data. DC performed the RNA-Seq analysis of the pathways. CL designed and performed the experiments, analyzed the data and critically revised the manuscript. MM conceptualized the project and provided resources for project completion. All authors read and approved the final manuscript.

## Conflict of Interest Statement

The authors declare that the research was conducted in the absence of any commercial or financial relationships that could be construed as a potential conflict of interest.

## Footnotes

1 https://www.ncbi.nlm.nih.gov/geo/query/acc.cgi?acc=GSE104527
