# Supplementary material for: Gene-Expression Analysis Identifies IGFBP2 Dysregulation in Dental Pulp Cells From Human Cleidocranial Dysplasia
Source: Front Genet. 2018 May 23;9:178. doi: 10.3389/fgene.2018.00178 (PMC5974155; doi:10.3389/fgene.2018.00178)
Supplement: Supplementary file 1 [file Data_Sheet_1.DOCX]

**Gene-expression Analysis Identifies IGFBP2 Dysregulation in Dental Pulp Cells from Human Cleidocranial Dysplasia**

Stephen Greene ^1,2^, Olga Mamaeva^2^, David K. Crossman^3^, Changming Lu^2,#^, Mary MacDougall^4,#^

**Supplemental Figure 1. Upstream gene targets of RUNX2.** Upstream gene targets of RUNX2 were identified and mapped using Ingenuity. Red and green colors indicate up- and down-regulated genes, respectively, in the CCD-011 sample.

**Supplemental Figure 2. Downstream gene targets of RUNX2.** Downstream gene targets of RUNX2 were identified and mapped using Ingenuity. Red and green colors indicate up- and down-regulated genes, respectively, in the CCD-011 sample.

**Supplemental Figure 1**


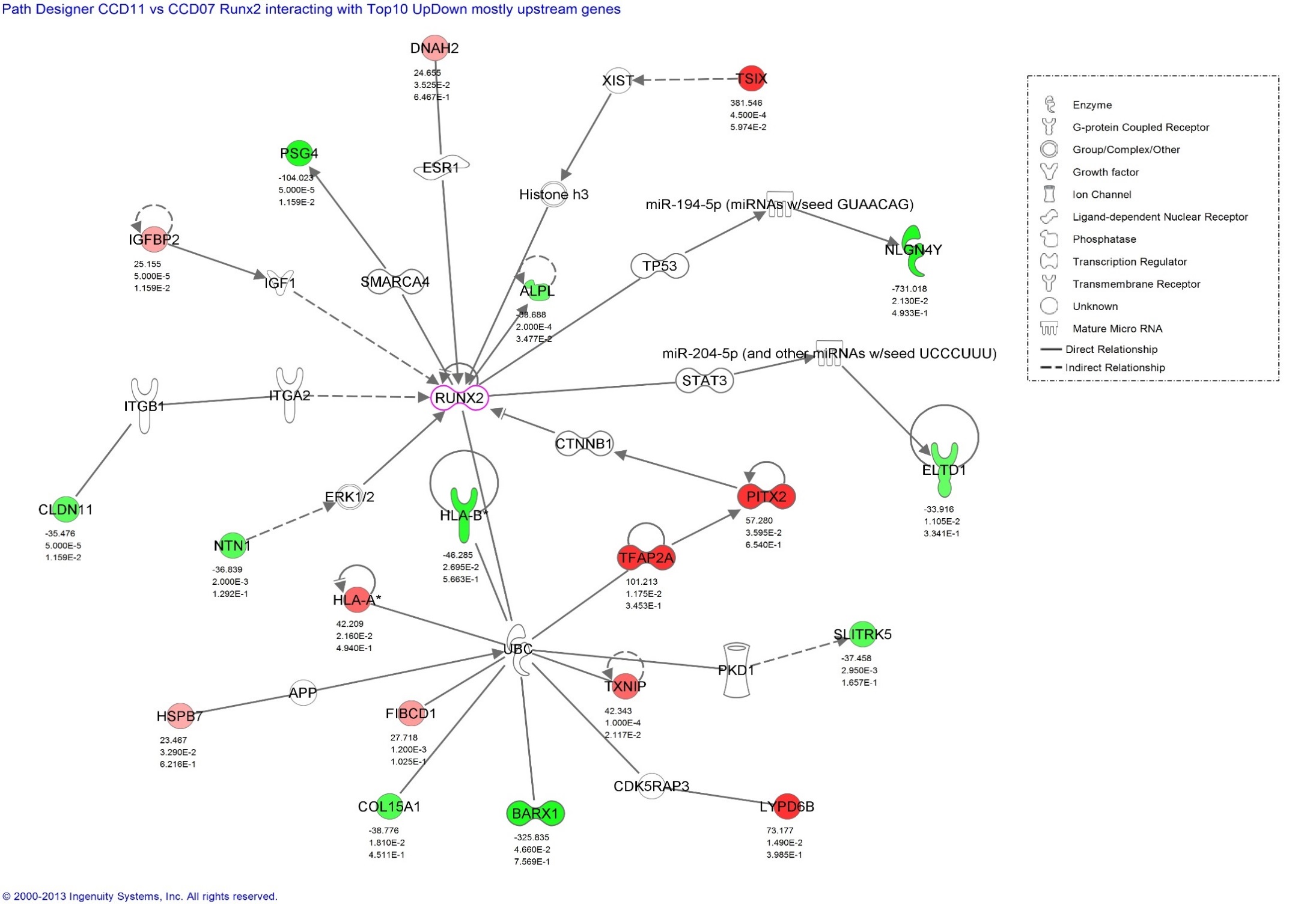


**Supplemental Figure 2**


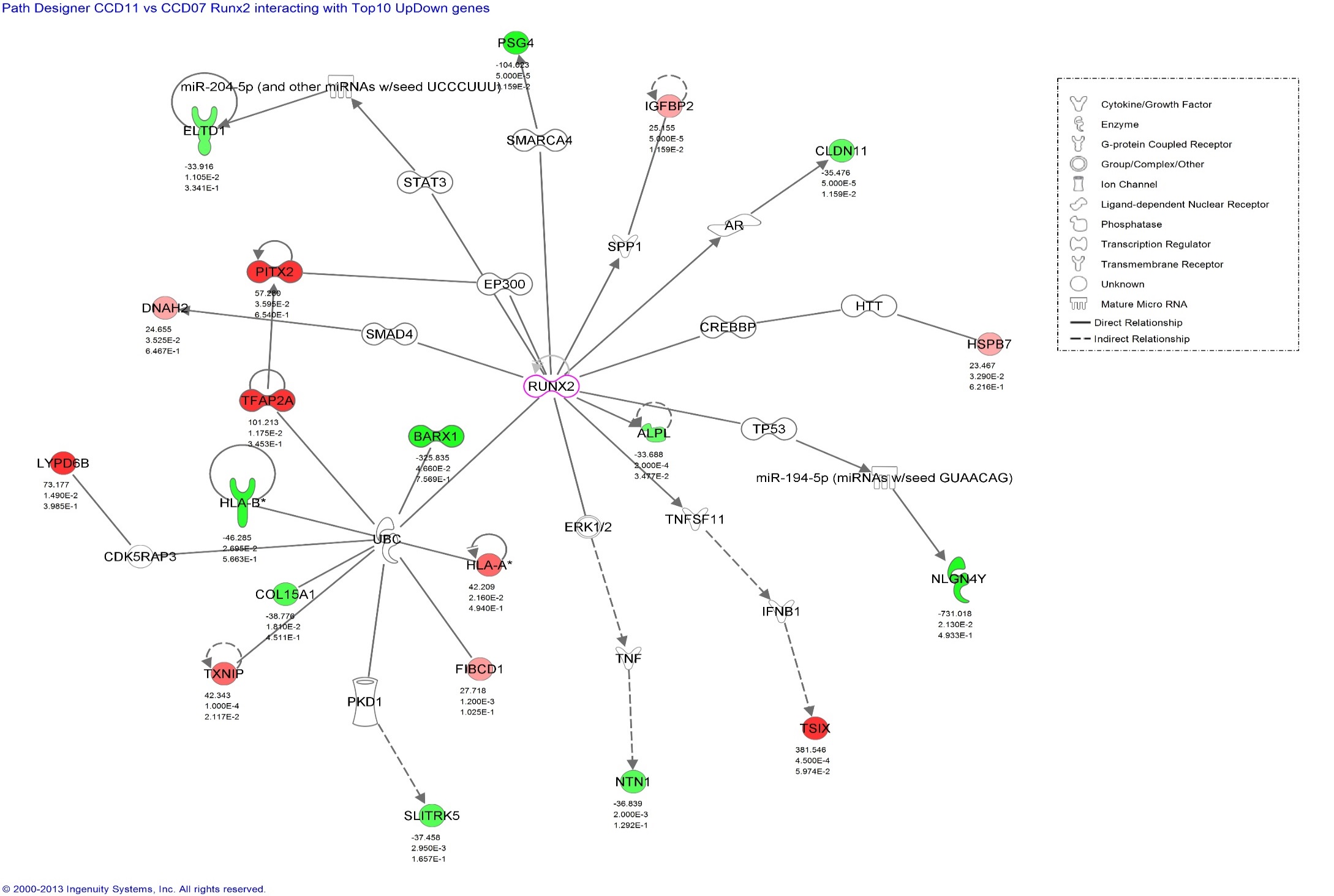


**Supplemental Table 1**. Isoform expression change in CCD-011 dental pulp cells

| **Reference ID** | **Gene** | **Locus** | **Fold Change** |
| --- | --- | --- | --- |
| NM_002780 | PSG4 | chr19:43696853-43709790 | -98.55 |
| NM_005602 | CLDN11 | chr3:170136652-170152479 | -42.48 |
| NM_001964 | EGR1 | chr5:137801180-137805004 | -16.38 |
| NM_012445 | SPON2 | chr4:1160720-1202750 | -10.37 |
| NM_000104 | CYP1B1 | chr2:38294745-38303323 | -8.86 |
| NM_000557 | GDF5 | chr20:34021148-34026027 | -7.87 |
| NM_058179 | PSAT1 | chr9:80912058-80945009 | -7.12 |
| NM_005542 | INSIG1 | chr7:155089485-155102004 | -6.04 |
| NM_001039349 | EFEMP1 | chr2:56093096-56151298 | -5.72 |
| NM_002852 | PTX3 | chr3:156977531-157221415 | -5.37 |
| NM_003638 | ITGA8 | chr10:15555953-15762353 | 4.51 |
| NM_006461 | SPAG5 | chr17:26904582-26926056 | 4.78 |
| NM_014220 | TM4SF1 | chr3:149086804-149095568 | 4.93 |
| NM_006739 | MCM5 | chr22:35796115-35820495 | 5.07 |
| NM_018685 | ANLN | chr7:36363758-36493400 | 5.07 |
| NM_001255 | CDC20 | chr1:43824625-43828873 | 5.42 |
| NM_001067 | TOP2A | chr17:38544772-38574202 | 5.43 |
| NM_003258 | TK1 | chr17:76170159-76183285 | 5.53 |
| NM_002961 | S100A4 | chr1:153516094-153518282 | 6.72 |
| NM_001122659 | EDNRB | chr13:78469615-78549674 | 7.68 |
| NM_002781 | PSG5 | chr19:43671894-43690688 | 8.13 |
| NM_002167 | ID3 | chr1:23884420-23886285 | 9.41 |
| NM_000962 | PTGS1 | chr9:125132808-125157989 | 9.45 |
| NM_004484 | GPC3 | chrX:132669775-133119673 | 12.38 |
| NM_005380 | NBL1 | chr1:19923470-19984949 | 14.30 |
| NM_002165 | ID1 | chr20:30193085-30194317 | 14.61 |
| NM_000930 | PLAT | chr8:42032235-42065194 | 16.19 |
| NM_001135690 | PENK | chr8:57353512-57359282 | 17.13 |
| NM_000597 | IGFBP2 | chr2:217498126-217529158 | 25.15 |
| NM_006472 | TXNIP | chr1:145438461-145442628 | 42.34 |
